# Development of a translational research pathway at the David Geffen School of Medicine University of California, Los Angeles

**DOI:** 10.5116/ijme.59ae.c221

**Published:** 2017-09-22

**Authors:** Lyudmyla Demyan, Christina Harview, Emily Miller, LuAnn Wilkerson, Isidro B. Salusky

**Affiliations:** 1 David Geffen School of Medicine, the University of California, Los Angeles, USA

**Keywords:** Research pathway, David Geffen School of Medicine, University of California, Los Angeles

## Introduction

Many standard medical curricula do not provide an environment that nurtures the diverse academic and professional interests of medical students, leaving few opportunities for curricular individualization.^1^ In response, the David Geffen School of Medicine (DGSOM) at the University of California Los Angeles (UCLA) has created a voluntary and mentored Pathway in Clinical and Translational Research for medical students interested in translational and clinical research. Several studies have shown that, when compared to voluntary projects, required extracurricular projects were not as beneficial for medical student learning and did not produce a successful outcome.^2^^,^^3^^,^^4^^,^^5^  The majority of the graduating students who have participated in the pathway since its creation six years ago found the pathway to be “very valuable” in the development of their scientific and medical careers.  Additionally, students who completed the pathway have published an average of 3.3 peer-reviewed publications per student, which suggests that optional research pathways may be a promising way to individualize medical school curricula. In this manuscript, we describe how the research pathway was established, how students perceived it, and ideas for improvements in the future. We hope that other medical schools will be able to use the information we provide here to initiate their own optional research pathways within their curriculum.

### Establishment of the research pathway

In an effort to make our medical education more individualized, the Dean's office at the David Geffen School of Medicine at UCLA utilized a National Institute of Health (NIH) grant dedicated to improving the quality of training in clinical and translational research in order to create a pathway that empowers students to pursue their passion in science without adding additional years to their training towards becoming physicians. The goals of the Pathway in Clinical and Translational Research are to: (1) utilize the UCLA research community to personalize education for students that are interested in the different aspects of research without amending the mainstream curriculum, (2) provide an opportunity for students to develop professional skills outside of the classroom, (3) allow the medical students to become a member of a research team, and (4) serve as a model curriculum with measurable outcomes.

A research pathway curriculum committee was established to initiate the development of a program of didactic seminars and clinical electives which when coupled with involvement in a research project that would lead to an official designation that will be listed on the student’s transcript and resume.

Required coursework includes: Introduction to Biomedical Research, a one year research preceptorship, the National Institute of Health Introduction to the Principles and Practices in Clinical Research Webinar, participation in a fourth-year society for career preparation that focuses on academic medicine, a poster presentation at any regional or national conference, and presentation of clinical or translational research at our fourth year medical student scholarship day.

The director of the program interviews each medical student interested in the pathway to identify specific areas of research interest, possible mentors, overall goals and meetings at regular intervals.  The student may already have a research mentor in mind, but often the director suggests specific mentors based on a student’s specific area of interest. Our research pathway model allows the medical school to add personalized and longitudinal research training by offering educational opportunities for students in various areas such as bioinformatics, clinical research design, and ethics in clinical research. Enrollment into the pathway is voluntary, and the choice of didactics and research focus is individualized.

### Outcomes

Upon entry into the program, first and second-year medical students complete a survey to self-assess their current knowledge and skills in various research topics and to assist them in identifying their expected outcomes of training in the Pathway in Clinical and Translational Research. The needs assessment begins the process of individualized study and mentoring. To measure the efficacy of the program, all participants complete the same survey prior to graduation. The entrance survey was designed to assess which research skills medical students already feel confident with and which skills they are most interested in developing. This information is critical in identifying the UCLA resources students need in order to develop their research skills. With the first two graduating classes completed, we now have a measure of how their confidence in these skills changed during their time in the pathway.  The assessment of the pathway’s utility can be used to gauge student confidence in the categories that they are most interested in developing.

An additional outcome measurement is the generation of peer-reviewed publications during medical school.  Of the 19 medical students that successfully completed the CTSI Medical Student Pathway to date, 13 have collaborated on and published a total of 62 publications (approximately 3.3 publications per student completing this program). In contrast, a study from the Mayo Medical School in Minnesota, USA, showed that 998 students participated in a required research thesis over a 26-year span and that they collectively published 554 research papers and 258 abstracts. This averages out to approximately 0.81 publications per student.^6^

Upon exit, students were also asked about the value of the pathway in the development of their goals as physicians.  Almost all of the graduates agreed that the Pathway in Clinical and Translational Research enabled them to participate in various stages of research.  They also agreed that, as a result of the completion of their training in the pathway, they plan to incorporate clinical and/or translational research into their careers as clinicians. The majority of the students also agreed that the longitudinal research experience improved their skills in critical appraisal of medical literature. The majority of surveyed students found that being part of the pathway was “very valuable” in finding a mentor, publishing, and presenting at various conferences.

In addition to training future doctors that may pursue academic medicine and become well-equipped clinicians, the Pathway in Clinical and Translational Research has served as a platform to support student-initiated ideas that focus on improving research opportunities. For instance, a small group of current medical students in the pathway have organized events to facilitate networking between medical students and the research community at UCLA. These same students have been deeply involved in the writing of this very publication.

## Conclusions

The Pathway in Clinical and Translational Research has been in place for the past seven years, and during this time four classes have graduated from the program. Currently, more than 100 students are enrolled in the program. Students have found that this pathway creates an individualized curriculum based on their interests and advances their academic growth and success. Based on what we have learned during the evolution of this program, future steps we would like to take include making the pathway more robust and student-centered and increasing opportunities for inter- and intra- professional collaboration in conducting research. Specifically, our goal is to expand the database of mentors to provide students opportunity to conduct research in various medical fields. In addition, student funding for dedicated research would allow students to dedicate more time for their project, such as taking a semester or a year off during medical school education.  Most medical school curriculums provide few opportunities for individualized academic development and our specialized pathway as we have described will allow students to advance academically in the area of their interest without substantial changes to the main curriculum.

### Acknowledgements

This research was partially supported by NIH National Center for Advancing Translational Science (NCATS) UCLA CTSI grant number UL 1TR001881.

### Conflict of Interest

The authors declare that they have no conflict of interest.
